# Advisor™ HD Grid Use in the Setting of Papillary Muscle Ventricular Tachycardia

**DOI:** 10.19102/icrm.2021.120103S

**Published:** 2021-01-15

**Authors:** Eathar Razak, Nasir Shariff, Gopi Dandamudi, Jason Clark

**Affiliations:** ^1^St. Joseph Medical Center, Tacoma, WA, USA; ^2^Abbott, Chicago, IL, USA

**Keywords:** Advisor HD Grid, idiopathic ventricular tachycardia, papillary muscle

A 63-year-old female with a history of nonischemic cardiomyopathy and systolic heart failure presented with frequent premature ventricular complexes and ventricular tachycardia (VT). A 12-lead electrocardiogram (right bundle branch block, II/III discordance, R/S V6 < 1) suggested the anterolateral papillary muscle as the target location. VT matching the documented clinical morphology was easily inducible and sustained at a cycle length of 355 ms.

The Advisor™ HD Grid Mapping Catheter, Sensor Enabled™ was used together with the EnSite™ Precision cardiac mapping system to create an ultra-high density (30,948-point) map while building geometry using the OneMap and Automap tools. A ViewFlex™ Extra ICE Catheter was used to visualize both papillary muscles and confirm accurate papillary geometry. This enabled the map to accurately project points (using the “nearest” algorithm) to the papillary muscles. The high-density grid frequently acquires timing/voltage data that cannot be duplicated by any other catheters; however, these signals of interest can be overridden with higher voltage signals using the “best duplicate” algorithm. Examining duplicates in the area of interest revealed a highly abnormal signal containing 281 ms of the cycle length spanning all of diastole. This point was located in the earliest identified area on the superior aspect of the anterolateral papillary muscle and unipolar signals showed an early QS deflection in that region. A TactiCath™ SE ablation catheter with a DF curve was placed on the area of interest. The signals seen on the high-density grid were not reproducible on the ablation catheter, but the decision was made to deliver a lesion at that location. Using 50 W/40°C with nominal pump parameters, the VT terminated **(Figures 1 and 2)** 0.7 seconds into the first burn and was noninducible after the first burn had been completed. Five additional lesions were delivered in the area of interest; 35 minutes after access was obtained, the procedure was concluded.

## Figures and Tables

**Figure 1:**
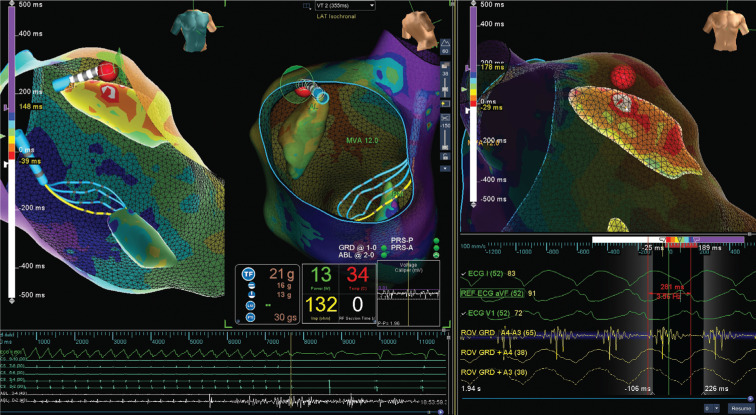
Ablation location, duration (< 1 second), and power (13 W, ramping up to 50 W) parameters when VT was terminated. The image on the right shows the Advisor™ HD Grid signal targeted for ablation. **Video 1** shows map creation, rapid identification of the ablation target, and the termination depicted in **Figure 1**.

**Figure 2:**
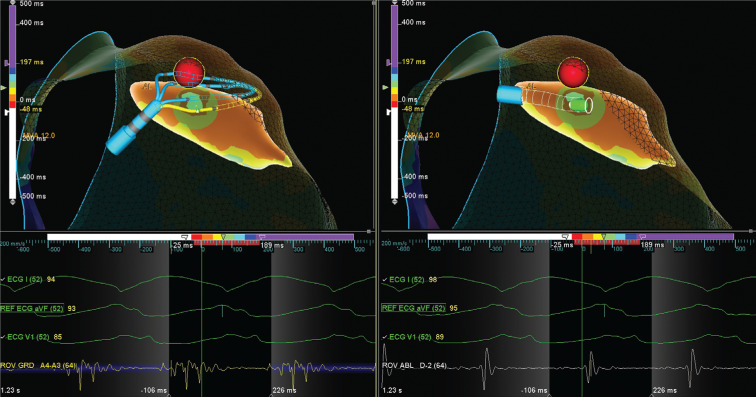
Advisor™ HD Grid signals in comparison with ablation catheter signals at the site of termination prior to ablation.

**Video 1.** Map creation, rapid identification of the ablation target, and termination depicted in **Figure 1**.

